# Exploitation of Bio-Inspired Classifiers for Performance Enhancement in Liver Cirrhosis Detection from Ultrasonic Images

**DOI:** 10.3390/biomimetics9060356

**Published:** 2024-06-14

**Authors:** Karthikamani Ramamoorthy, Harikumar Rajaguru

**Affiliations:** 1 Department of ECE, Sri Ramakrishna Engineering College, Tamil Nadu 641022, India; karthikamaniece@gmail.com; 2 Department of ECE, Bannari Amman Institute of Technology, Tamil Nadu 638401, India

**Keywords:** PCM, FCM, PFCM, sample entropy, GMM, SDC, AAO, SVM

## Abstract

In the current scenario, liver abnormalities are one of the most serious public health concerns. Cirrhosis of the liver is one of the foremost causes of demise from liver diseases. To accurately predict the status of liver cirrhosis, physicians frequently use automated computer-aided approaches. In this paper, through clustering techniques like fuzzy c-means (FCM), possibilistic fuzzy c-means (PFCM), and possibilistic c means (PCM) and sample entropy features are extracted from normal and cirrhotic liver ultrasonic images. The extracted features are classified as normal and cirrhotic through the Gaussian mixture model (GMM), Softmax discriminant classifier (SDC), harmonic search algorithm (HSA), SVM (linear), SVM (RBF), SVM (polynomial), artificial algae optimization (AAO), and hybrid classifier artificial algae optimization (AAO) with Gaussian mixture mode (GMM). The classifiers’ performances are compared based on accuracy, F1 Score, MCC, F measure, error rate, and Jaccard metric (JM). The hybrid classifier AAO–GMM, with the PFCM feature, outperforms the other classifiers and attained an accuracy of 99.03% with an MCC of 0.90.

## 1. Introduction

Improvement of the computer-aided diagnosis system is essential in the medical field because it can provide an objective and systematic second opinion to the patients [1]. There are various image modalities used for disease diagnosis, such as MRI, X ray imaging, computer tomography, and ultrasound. Clinicians prefer ultrasound imaging due to its non-invasive and cost-effective nature, with no ionizing radiation effects [2]. Sound waves, instead of radiation, are used to produce internal body structure images of, for example, the uterus, the liver, blood vessels, and the kidney. Ultrasound imaging is the most effective and safest method by which to diagnose the abnormalities in abdomen [3]. In humans, the liver is a large and extremely important organ; it is involved in metabolism, which produces protein, cholesterol, and bile acid, among other things. The liver’s primary function is to filter blood from the digestive tract before it is distributed to the rest of the body. There are two types of liver diseases: cyst and cirrhosis [4]. Liver cirrhosis is a chronic and progressive liver disease characterized by the irreversible scarring and fibrosis of the liver tissue. It is a consequence of various chronic liver conditions and is often considered the end-stage of liver damage. Liver cirrhosis is a significant global health issue and can lead to severe complications, including liver failure, portal hypertension, and increased risk of liver cancer. Liver cirrhosis often develops gradually, and in the early stages, it may not present noticeable symptoms. However, as the disease progresses, individuals may experience symptoms such as fatigue, weakness, loss of appetite, weight loss, abdominal pain, jaundice (yellowing of the skin and eyes), easy bruising or bleeding, and fluid retention in the abdomen or legs. Among many of the numerous liver disorders that can lead to cirrhosis, some progress quickly, while others progress more slowly [5]. Based on data gathered from Cancer Incidence Statistics, Globocan 2020, the estimated prevalence of cirrhosis in the world’s population is 4.7% [6]. Hepatic steatosis is the abnormal addition of fat in the liver and hepatic cells [7]. Steatosis can be temporary or permanent and can lead to a variety of problems, including liver cirrhosis. Steatosis is a reversible condition that can be resolved through behavioral changes. Early detection of liver cirrhosis is critical as there is a chance of saving the liver from serious complications [8]. The abnormality in focal diseases is confined to a small area of liver tissue; whereas in diffused diseases, the abnormality is scattered throughout the liver volume [9]. Liver cancer is a common malignant disease throughout the world, particularly in Southeast Asia and Sub-Saharan Africa. With 500,000 people affected each year, liver cancer is the sixth most common cancer in the world. Globally, the number of people diagnosed with liver cancer is increasing [10]. Every year in India, almost 10 lakh new cases of liver cirrhosis are reported. The World Health Organization ranks liver disease as the tenth leading cause of death in India [11]. It is important to detect diseases in the early stage. In this paper, liver diseases are detected and classified through known clustering methods and nature-inspired classifiers from the ultrasonic image database.

The ultrasonic imaging technique is the widely used imaging modality to diagnose the patient’s disease condition because it is safe and economical. Various techniques are proposed to diagnose liver disease by the researcher. Nasrul Humaimi Mahmood et al. (2012) [12] suggested a method of enhancing the diseased liver region using watershed segmentation. The ultrasound images are preprocessed, and the background markers are computed using the thresholding operation to clean up the pixels which do not belong to any object. The segmentation function is modified to obtain minimum foreground and background marker locations. After this, the watershed transform of the segmented function is calculated to highlight the diseased region of liver image. 

Nishant Jain et al. (2016) [13] presented a method known as iterative FCM (IFCM), which can be applied to fragment a focal liver lesion from ultrasound images of the liver. The n cluster FCM method results in a distribution of centroids that is not uniform; whereas the IFCM method results in centroids that are evenly distributed. The authors compared IFCM accuracy with that of the edge-based active contour Chan–Vese (CV) method and the maximum a posteriori Markov random field (MAP-MRF) method. The proposed model provides the highest accuracy (99.8%). Bharath et al. (2017) [14] proposed a method for differentiating the texture feature of a fatty liver that is based on the curvelet transform and the SVD. The gradient, as well as the curves in the texture, can be improved with the help of the curvelet transform. The curvelet decomposed image is used as a source for the extraction of the SVD features. The classification is performed by the cubic SVM classifier. The proposed technique successfully classified liver fat into four classes, with an accuracy of 96.9%. Rajendra Acharya et al. (2016) [15] proposed curvelet transform and entropy feature to classify cirrhosis in ultrasonic images. The HOS phase, fuzzy, Reyni, Shannon, Kapoor, Vajda, and Yager entropy features are extracted from CT coefficients. The probabilistic neural network classifier is used for classification. The probabilistic neural network (PNN) classifier can distinguish between a normal liver, fatty liver disease (FLD), and cirrhosis with 97.33% accuracy using the proposed method. Rajendra Acharya et al. (2016) [16] proposed radon transform and DCT for extracting the features for normal and fatty liver images. The images are classified using decision tree, K-NN, PNN, SVM, Fuzzy Sugeno, and Adaboost classifier and attained higher accuracy. 

Wang, Q. et al. (2017) [17] proposes a method for liver cirrhosis detection based on texture analysis and ensemble clustering. Texture features are extracted from liver ultrasound images, and K-means clustering and spectral clustering are applied to partition the feature space. The ensemble clustering results are then used for classification. Saranya, K et al. (2019) [18] present a liver cirrhosis diagnosis method using K-means clustering and SVM classification. Features are extracted from liver ultrasound images, and K-means clustering is applied to group the feature vectors. The SVM classifier is used for cirrhosis diagnosis based on the clustered features.

A growing number of application domains are utilizing machine learning and deep learning techniques due to their ability to efficiently address a broad range of challenging issues. To improve the prediction rate of liver abnormalities the feature extraction is based on sparse K-SVD and rigid regression mathematical models can be used in future work. For SVM and GMM, classifiers, hyper parameters selection is undertaken through ADAM and RanADAM search, which further enhance the classification performance. Using models pre-trained on large datasets like ImageNet and fine-tuning them on medical imaging datasets can significantly reduce the need for extensive labeled data and improve performance. Deep learning (DL) uses hierarchical architectures to automatically learn high-level abstractions from data. The most popular DL techniques are based on neural networks. Modern CNN architectures, such as ResNet, DenseNet, and EfficientNet, provide improved accuracy and robustness in image classification tasks. For volumetric data analysis, 3D CNNs can be used to analyze three-dimensional ultrasound images, capturing spatial features more effectively than 2D CNN [19]. 

Generative adversarial networks (GANs) can be used for super-resolution imaging, enhancing the quality of low-resolution ultrasound images and making subtle features more detectable, generating automated diagnostic reports based on ultrasound analysis, and reducing the administrative burden on radiologists. Current advancements in machine learning and deep learning techniques are significantly enhancing computer-aided diagnosis (CAD) systems for the early detection and classification of liver diseases using ultrasonic imaging [20].

This paper is structured in the following manner. The preprocessing and feature extraction techniques are discussed in section two, while the third section deals with the classifiers, namely, GMM, SDC, harmonic search, SVM (linear, polynomial, RBF), AAO, and the hybrid classifier: AAO with GMM. The results and discussions are explained in the fourth section. The paper is concluded in the fifth section.

## 2. Materials and Methods

In terms of global mortality, cancer is the second leading cause. When compared to more traditional image processing approaches, ultrasonic imaging seems to be a non-invasive and vital tool for liver cancer identification and categorization. Therefore, ultrasonic imaging techniques are used in this study. The primary goal of these studies is to improve liver cancer diagnosis by providing more precise classification systems. Accurate classification of cancer helps patients to receive the care they need at a lower cost and also reduces their risk of developing the disease. There is a tendency for classifier accuracy to decrease due to the presence of unimportant and noisy images in the captured image. The effective filtering method is used to remove the unwanted signal in ultrasonic images

**Figure 1:**
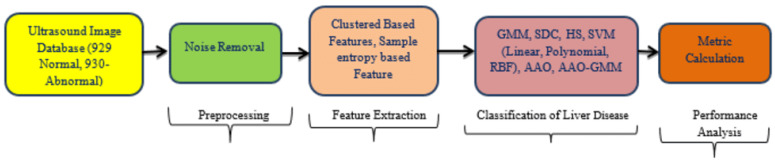
Schematic for ultrasonic image-based liver abnormality detection.

**Figure 2:**
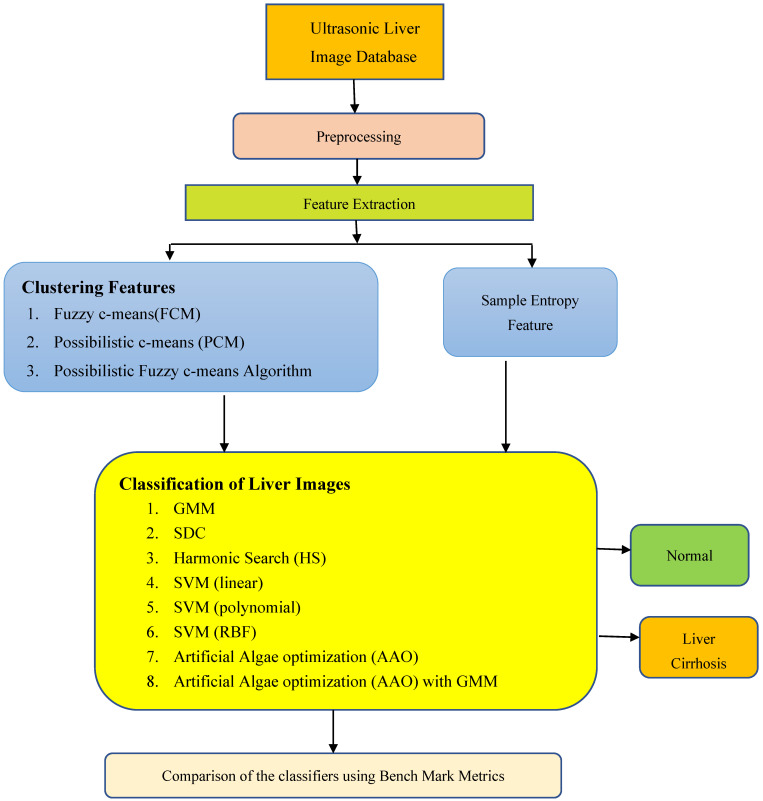
Detailed work flow for liver abnormality detection.

The overall schematic representation for detecting liver cirrhosis from ultrasound images is shown in Figure 1, and the detailed workflow is illustrated in Figure 2. This article explores a group of 1859 ultrasound images from the Cancer Imaging Archive (929 normal, 930 abnormal). Each picture measures around 790 by 960 pixels. The images are preprocessed before feature extraction. The FCM, PCM, FPCM, and sample entropy algorithms are employed to extract the significant features from the images and given as input to the classifiers. In total, eight classifiers are employed in this work. The MATLAB 2014a software is used to carry out this investigation [21]. The performance of classifiers is evaluated and discriminated using parameter metrics for cluster-based features and the sample entropy feature.

### 2.1. Preprocessing

**Figure 3:**
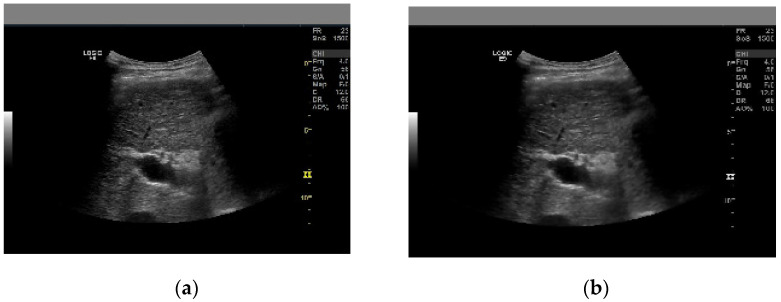
Illustration of sample ultrasonic liver cirrhosis images: (**a**) original image; (**b**) adaptive wiener filtered image.

Speckle noise is the main thing that changes the usefulness of ultrasonic images. The speckle noise affects the contrast and resolution of the images. So, an effective noise filtering technique will be utilized for the elimination of noise, and adaptive Wiener filtering is used in this work to suppress the noise [22]. In adaptive Wiener filtering, the filter’s output is modified based on the image’s local variation. The final objective is to achieve the smallest possible mean square difference between the restored and original images. This method is superior to other filters in terms of filtering effect, and it is especially helpful for conserving the image’s edges and high-frequency regions [23]. The equation below represents the speckle noise model:

(1) g(q,r) = h(q,r) + m(q,r),

where *g*(*q,r*) represents the noisy image, the noise-free image is denoted is represented by *h*(*q,r*), and the speckle noise component is denoted by *m*(*q,r*) [22]. In adaptive Wiener filtering, the filter’s final result is adjusted according to the image’s local variation. To some extent, the adaptive filter can fine-tune itself. Automatically adjusting to different input signals, it improves image restoration quality by minimizing the mean square error. The peak signal-to-noise ratio (PSNR) and mean square error (MSE) are the metrics used to select filters. The PSNR and MSE of the adaptive wiener filter are 31.3119 db and 48.0713, respectively. A higher PSNR value indicates that more noises have been removed, while a lower MSE indicates less error. The adaptive Wiener filter performs better at noise reduction, with a minimum MSE value of 48.0713. When compared to other filters, this one has a better filtering effect, and it works great for keeping the image’s edges and high-frequency areas intact. Figure 3 below shows the original and filtered ultrasonic images. 

### 2.2. Clustering Methods for Feature Extraction 

The function of feature extraction is to convert raw data input into numerical features. Typically, images are represented as raw data. Its purpose is to extract characteristics that indicate meaningful information extraction from ultrasonic images. The preprocessed and noise-removed ultrasound images are further processed to extract the features by employing three clustering algorithms and one entropy feature, namely, possibilistic fuzzy c means (PFCM), fuzzy c means clustering (FCM), and possibilistic c-means (PCM) techniques, as well as sample entropy. In order to expedite the embedded distinct properties of the liver image, the classes are analyzed via statistical variables like mean, variance, skewness, kurtosis, and Pearson correlation coefficient (PCC), as shown in Table 1.

As observed from Table 1, PCM features exhibit poor values of mean and variance among the classes, and skewness is one-sided in nature, along with tailed kurtosis. The intra class PCC is identified with no correlation. The CCA represents higher correlation across the classes for all the feature extraction methods. As in the case of the FCM method, only PCC indicates slight improvement in the intra class correlation. PFCM features demonstrate positive skewness and tailed kurtosis with moderate PCC for intra classes. The sample entropy features depict negative skewness and flat kurtosis with improved PCC for intra classes. As to the statistical parameters of the extracted features through four different methods, Table 1 clearly demonstrates the non-Gaussian, overlapping, and skewed nature of the features, as well as less correlation.

#### 2.2.1. Fuzzy c-Means Clustering (FCM)

One of the most basic methods of fuzzy clustering is fuzzy c-means (FCM). This process of adaptation converges to a stationary state or local minimum. The FCM method is used to examine datasets that are connected through both Euclidean and non-Euclidean distances [24]. In FCM, the data point may be a member of multiple clusters simultaneously, and this is known as the membership function. Based on the similarity, the membership value is assigned to a data sample, and normally, the value lies between 0 to 1. The optimum solution is obtained by continuously updating the cluster center and membership function. The updated values are obtained by solving the cost function [25].

Let Y = {y_1_, y_2_, y_3_, …, y_N_}, where N represents the data sample. It is divided into “*c*” clusters by reducing the cost function.

(2) $$K\;=\;{\sum_{l=1}^{M}\sum_{m=1}^{c}{w_{lm}}^{n}\;\left\|y_{l}\;-\;u_{m}\right\|}^{2}$$

For the *m*th cluster, *w_lm_* represents the membership of *y_l_*. The *m*th cluster is represented by u_m_, and $\left\|\;.\;\right\|$ represents norm metric [26].

#### 2.2.2. Possibilistic c-Means (PCM) Algorithm

PCM is another clustering technique developed by Keller in 1997. This approach is suitable for unwinding compact clusters [27]. The approach is distinguished from previous clustering algorithms in that the resultant subdivision of the data can be considered as a probabilistic partition, and the membership values can be considered as degrees of possibility of the points belonging to the classes. A scalar v_ij_ can be used to associate each data vector y_i_ with a cluster Cj [28].

Consider an unnamed dataset Y = {y_1_, y_2_, y_3_, …, y_n_}$\subset \;P^{r}$_._ The objective function is minimized to determine the partitioning of Y into 1 < c < m fuzzy subsets.

(3) $$K_{m}\;(E,F)\;=\;\sum_{a=1}^{c}\sum_{b=1}^{m}{v_{ab}}^{n}\;{F_{ab}}^{2}\;+\;\sum_{a=1}^{c}\sum_{b=1}^{m}{\left(1-v_{ab}\right)}^{m}$$

Here, F_ab_ = $\left\|y_{b}-\;v_{a}\right\|$, and the number of clusters are given as “*c*”, and the number of data points is denoted as *m*. The positive constant $\eta_{a}$ is given by

(4) $$\eta_{a}\;=\;\beta \;\frac{\sum_{a=1}^{n}{v_{ab\;}}^{m}\;{F_{ab}}^{2}}{\sum_{b=1}^{n}{v_{ab\;}}^{m}}\;,\beta >0.$$

The value of $\eta_{a}$ is always 1 for all values of *a*,*b*. $F_{a,b}\;=\left\|y_{a}\;-\;v_{b}\right\|\;>\;0$. $\underset{\left(E,F\right)}{Min}\;K_{m}\;(E,F)$ is improved, and the following PCM method is derived [29]

(5) $$v_{ab}\;=\;{\left[1+{\left(\frac{{F_{ab}}^{2}}{\eta_{a}}\right)}^{\frac{1}{m-1}}\;\right]}^{-1}\;,\;\forall a,b,$$

(6) $$v_{ab}\;=\;{\left[1+{\left(\frac{{F_{ab}}^{2}}{\eta_{a}}\right)}^{\frac{1}{m-1}}\;\right]}^{-1}\;,\;\forall a,b.$$

#### 2.2.3. Possibilistic Fuzzy c-Means Algorithm (PFCM)

Outliers and noise are two of FCM’s greatest issues. To address this issue, the PCM algorithm is offered; however, it is sensitive to initial conditions and suffers from coincident clustering. As a hybrid of FCM and PCM, PFCM is introduced [30]. In noisy data, it is suggested that both hard and soft clustering use a norm parameter other than the Euclidean norm in order to improve FCM performance. The FCM algorithm finds the most compact clusters by diminishing the loss function [31].

(7) $$J(X,Y,Z;\;A)\;=\;\sum_{k=1}^{M}\sum_{l=1}^{c}(C_{FCM}{x_{kl}}^{\eta }+\;C_{PCM}{y_{kl}}^{\eta })\;{\left\|\vec{a_{k}}\;-\;\vec{z_{l}}\right\|}^{2}\;+\;\sum_{l=1}^{c}\gamma_{l}\;+\;\sum_{k=1}^{m}(1-Z_{kl}{)}^{\eta }$$

Here, $C_{FCM}\;\geq 0\;\&\;C_{PCM}\;\geq \;0$ denote the respective significance of the membership class $x_{kl},\;Z_{kl}$.

#### 2.2.4. Sample Entropy as a Feature

Entropy is a term that deals with prediction and uncertainty, with higher entropy levels always indicating a lack of system order and an increase in randomness. Sample-entropy-based feature extraction provides a valuable tool for quantifying the complexity and regularity of signals. It offers a means by which to extract informative features that capture important characteristics of the underlying dynamics. These features can be used for various analysis tasks, including classification, anomaly detection, and monitoring in different domains. Sample entropy is less susceptible to noise and can be used with short time series data. The negative logarithm of the conditional probability that two sequences identical with respect to m points persist, like at the next point is SampEnt (m,r,N). A maximum level usually indicates more irregularity or complexity in the dataset [32].

(8) $$SampEnt\;(n,p)\;=\;\underset{M\to \infty }{Lim}\left\{-\ln\left[\frac{C^{m}\;(p)}{D^{m}(p)}\right]\right\}$$

For finite number of data points the sample entropy is given as follows:

(9) $$SampEnt\left[p,r,N\right]\;=\;\ln\left[\frac{D^{m}(r)}{C^{m}(r)}\right].$$

**Figure 4:**
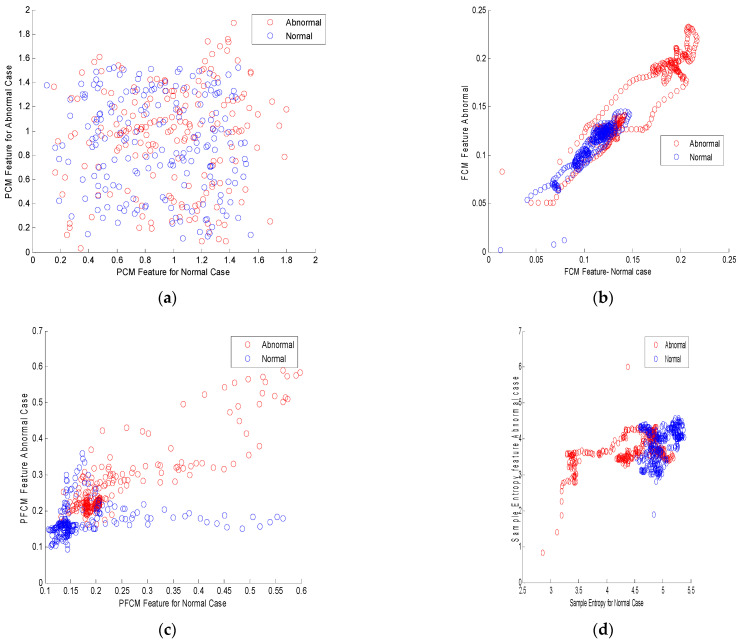
Illustrations of scatter plots for different feature extraction techniques: (**a**) scatter plot for PCM feature; (**b**) scatter plot for FCM feature; (**c**) scatter plot for PFCM feature; (**d**) scatter plot for sample entropy feature.

Figure 4a illustrates the scatter plot for normal and cirrhotic cases for the PCM feature. It clearly indicates the features are fully scattered with outliers. Figure 4b shows the scatter plot for FCM features, which indicates the overlapped and non-linear properties of the feature. The scattered plot for the PFCM feature is depicted in Figure 4c. It indicates that the normal and cirrhotic features overlap with outliers and are partially scattered. As to the scatter plot of the PFCM feature, it resembles a traditional XOR problem. Figure 4d shows a scatter plot for the sample entropy feature. This indicates that the features are non-linear, overlapped, and non-Gaussian. 

## 3. Bio Inspired Classifiers for Classification of Liver Cirrhosis from Extracted Features

The various bio-inspired classifiers are explored in this article for classification of liver abnormality, as explained below.

### 3.1. Gaussian Mixture Model (GMM) as a Classifier

A Gaussian mixture model (GMM) is a type of prediction model in which all data samples are formed from a combination of finite Gaussian densities [32]. The Gaussian mixture model (GMM) is a probabilistic model that represents a probability distribution as a weighted sum of multiple Gaussian distributions. Each Gaussian component in the mixture model represents a cluster or a mode in the underlying data distribution. GMMs have been widely used for various tasks, including clustering, density estimation, and data modeling. As a result, GMM uses a series of Gaussian densities to model the distribution of a data collection. Consider an n-dimensional sample space with random vector y obeying the Gaussian distribution; in this case, the PDF is given as [33]

(10) $$P(y)\;=\;\frac{1}{{\left(2\pi \right)}^{m/2}\;{\left|\sum \right|}^{1/2}}\;e{\;}^{\left(-1/2)\;{\left(y-\mu \right)}^{T}\;\sum^{-1}\;(y-\mu \right)},$$

where μ denotes the mean, and the covariance matric is ∑. The two parameters μ, ∑ define the Gaussian distribution. 

### 3.2. Softmax Discriminant Classifier (SDC)

The Softmax discriminant classifier’s objective is to accurately identify the class to which a test sample fits. This is accomplished by calculating the distance between both the training sample and the test sample of that particular class. Assume the training set $K\;=\;\left[K_{1},\;K_{2},\;\ldots ,\;K_{m}\right]\;\in \;{ℜ}^{a\times b}$ derives from “m” different classes, $K_{m}\;=\;[K_{1}^{m},\;K_{2}^{m},\;\ldots ,\;K_{bm}^{m}]\;\in \;{ℜ}^{a\times bm}$ denotes the “b_m_” samples from the mth class, where $\sum_{j=1}^{m}b_{j\;}\;=\;b$ [34]. We use the m class samples to find the test sample with the least amount of reconstruction error. The concept of SDC can be accomplished by increasing the value of non-linear conversion among the q class samples and the test sample. The SDC is given by

(11) $$g(k)\;=\;\arg\;\underset{j}{\max}\;Z_{k}^{j},$$

(12) $$g(k)\;=\;\arg\;\underset{j}{\max}\;\log\left(\sum_{i=1}^{d_{i}}\exp(-\lambda \;{\left\|k-k_{i}^{j}\right\|}_{2})\right).$$

The distance between the *j*th class and test sample is denoted by *g*(*k*). λ should be greater than zero to introduce a penalty cost. Hence, if K detects to the *j*th class, then *k* and $k_{i}^{j}$ would have same characteristic, ${\left\|k-k_{i}^{j}\right\|}_{2}$ is progressing class towards zero, and the maximum possible value is achieved by maximizing $Z_{j}^{k}$ in an asymptotic manner [35].

### 3.3. Harmonic Search Algorithm (HSA) as a Classifier

HSA is an evolutionary algorithm which is inspired by musicians’ improvising. This algorithm (HS) aims to mimic improvisational procedure used by musicians while presenting music. In the traditional way of improvising, to make wonderful music, musicians constantly fine-tune their instruments [36]. The fundamental stages of HS execution are as follows.

1. Parameters for the HS algorithm and problem configuration:

Here, the objective function f(k) is subject to $k_{i}\;\in \;K,i=1,2,3,\ldots ,M$, which is minimized or maximized using the optimization technique. A set of decision variables is represented by M; then, for each variable, the set of all possible variables is represented by K, i.e., $k_{iL}\;\leq \;K\;\leq k_{iv}$. The upper and lower boundaries for each decision variable are denoted by k_iL_ and k_iv_. To initialize the HS algorithm, the following parameters are considered:

1. Harmony memory size (SHM);
2. Harmony memory consideration rate (HMCR);
3. Pitch adjusting rate (PAR);
4. Bandwidth (BW);
5. Maximum number of iterations (Maxitr).

2. Initialization of Harmony Memory:

In the form of a matrix, all of the decision variables are saved in the memory of the system. The initial HM for the globally optimal solution is drawn from a uniformly distributed distribution of values within k_iL_ and k_iv_. 

3. New Harmony Development:

The following limitations are used to create a new harmony through the solution development phase.

- Consideration of memory;
- Pitch change;
- Random selection.

4. Harmony memory Updating:

We f the harmony vector fitness function. The new harmony vector should replace the old one if its fitness function is relatively low. Otherwise, we keep the old harmony vector.

5. Evaluate the ending criteria:

We repeat steps 3 and 4 followed until the maximum number of iterations achieved.

**Figure 5:**
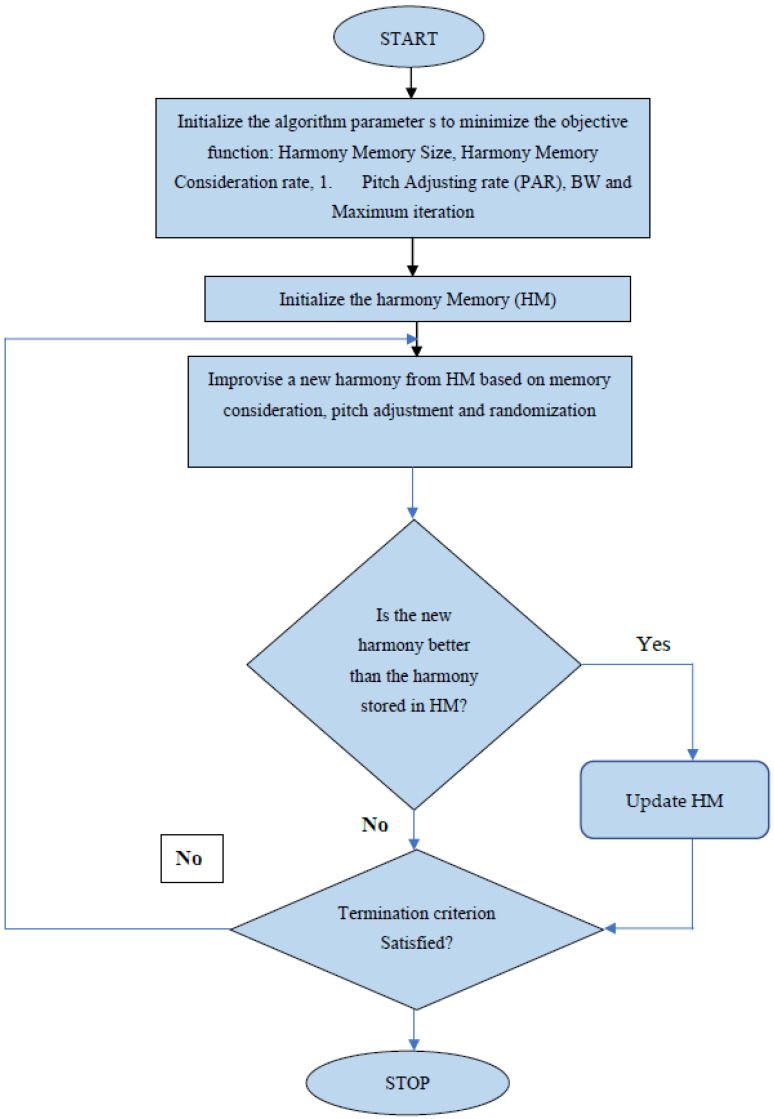
Flowchart of HS algorithm.

Figure 5 shows the cirrhosis classification. HS-based cirrhosis classification uses ultrasound liver images one by one. The normalized n mean feature is set as HM for classification; i.e., the harmony memory size is “n”; after this, the initialization harmonic search is started. The class as a whole will reach a state of perfect harmony when the maximum number of iterations is conducted. However, in the case of binary classification, exact harmony is not needed; thus, the highest number of iterations is picked as the stopping condition [37]. 

On the basis of learning by doing, the value of SHM was decided to be 16, and the matrix was set to 3. The limits of the decision variables k_iL_ is 1 and k_iv_ is 0.1. The BW and variation in pitch-control amount is selected as 0.1. The final stage of liver cirrhosis classification is depicted in Figure 5. The best harmony vector is taken as the result of the harmony search for each image, and the mean of the best harmony vector is then determined.

### 3.4. Support Vector Machine as a Classifier

One of the most powerful supervised learning algorithms is SVM. It is a binary classifier and a statistical learning method. For image classification, SVM is incredibly intelligent. It is utilized in the hyper plane to set decision boundaries, as well as to separate relevant and irrelevant vectors and to sort data points into different classifications [38]. In SVM, input data are transferred into higher dimensional feature space using kernel functions for multiclass problems. The direct acyclic graph, binary tree, one against one and one against all—these are some techniques employed to solve the multi-class SVM problem. Consider a feature “Z” with label “h” for a binary classifier; the class labels are represented as h ∈ (−1,1). Assume the parameters *y*,*a* instead of the classifier with vector θ; the classifier equation is given as

(13) $$g_{y,a}(z)\;=\;K(y^{T}z+a).$$

Here, k(x) = 1 if x≥0 and k(x) = −1 otherwise. The term “*a*” is separated from other parameters using *y*,*a*. In this work, we used SVM with three kernels (radial basis function (RBF), linear, polynomial) [39].

1. SVM with RBF (radial basis function) kernel:

(14) $$H(y_{i}\;,\;y_{j})\;=\;\frac{\exp(-\gamma {\left\|y_{i}\;-\;y_{j}\right\|}^{2}}{2\sigma^{2}}.$$

2. SVM with linear kernel:

(15) $$H(y_{i}-y_{j})\;=\;y_{i}^{T}\;y_{j}.$$

3. SVM with polynomial kernel with degree d:

(16) $$H(y_{i}-y_{j})\;={{\left(\;y_{i}^{T}\;y_{j}+1\right)}_{j}}^{d}.$$

### 3.5. Artificial Algae Optimization Algorithm (AAO)

In 2015, AAO was introduced as a population-based optimization technique. Algal colony optimization (AAO) is an attempt to model biological behavior in algae colonies in order to find efficient solutions to optimization problems with a repeated solution space [40]. A colony of algae is a collection of algal cells that live together. For example, when a single algal cell divides into two, both of the resulting cells continue to reside in close proximity to one another. An algal colony works similarly to a single cell; it changes in a clump, and cells in the colony may die if living conditions are inappropriate. An external force, such as shear, or other unfavorable conditions, may disperse the colony, with each dispersed section becoming a new colony as life progresses.

(17) $$Population\;A\mathrm{lg}al\;colony\;=\;\left[\begin{array}{ccc} y_{1}^{1} & y_{1}^{2}\;.\;. & y_{1}^{C}\\ y_{2}^{1} & y_{2}^{2}\;.\;. & y_{2}^{C}\\ . & . & .\\ . & . & .\\ y_{N}^{1} & y_{N}^{2}.\;. & y_{N}^{C} \end{array}\right],$$

where $y_{j}^{i}$ is an algal cell in the *i*th element of the *j*th algal colony. The evolutionary process, helical movement, and adaptability are the three phases of AAO [41].

Evolutionary Process:

The growth characteristics of the algae are defined by the evolutionary process. It can be modeled using the Monod equation:

(18) $$H_{j}^{t+1}\;=\;\left(\frac{e_{i}(y)}{L+e_{i}(y)}\right)\;*\;H_{i}^{t}\;,\;(i=1,2,3\ldots ,N).$$

The size of the algal colony is H_1_, and the number of algal colonies is represented by “*N*” [42].

Adaptation Process: 

An undeveloped algal colony will go through the process of adaptation in an effort to model itself after the most dominant algal colony in its surrounding atmosphere. This procedure is completed when the algorithm’s hunger level is changed. For every artificial algal cell, the initial starving value is zero. When algae are subjected to insufficient illumination, the starvation value increases as “*t*” grows [43].

(19) $$Starving^{t}\;=\;Max\;B_{j}^{T},(j=1,2,3,\ldots N)$$

(20) $${\mathrm{Straving}}^{t+1}\;=\;Starving^{t}+(biggest^{t}\;-\;Starving^{t})*rand$$

Helical Movement

To maximize their exposure to light, algal cells and colonies migrate to and reside near the water’s surface. Real-life algal cells move helically. Shear force (viscous drag) is proportional to algal cell size, and gravity is zero in AAA. Its model volume decides its size. As an effect, the hemisphere’s surface area is converted to a friction surface.

(21) $$T_{yj}\;=\;2\pi {\left(\sqrt[{3}]{\frac{3\;H_{j}}{\pi }}\right)}^{2}$$

**Figure 6:**
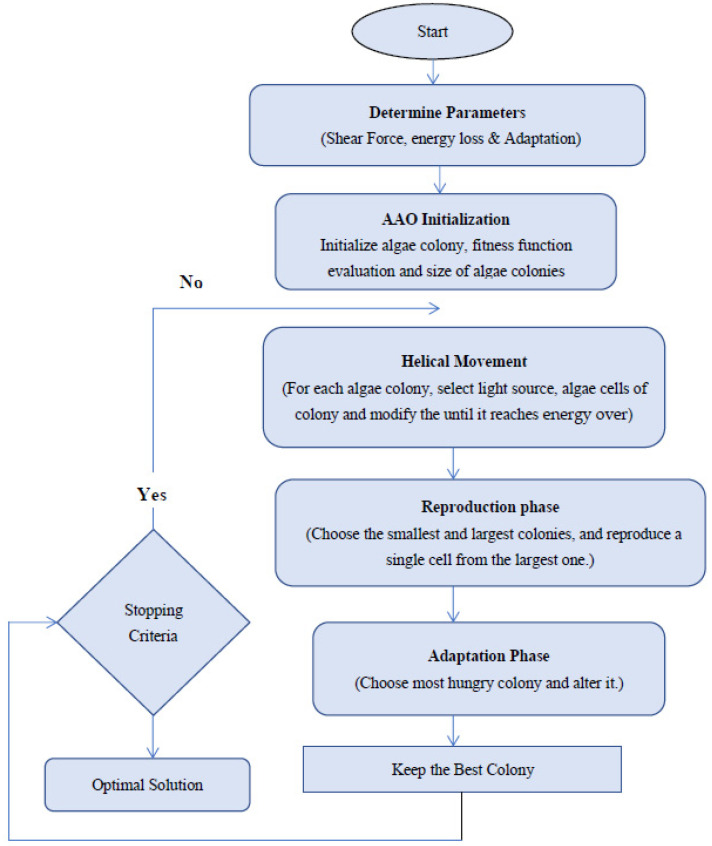
Flowchart of AAO algorithm.

The helical motion of an algal cell is based on a random process that occurs in all three planes [44]. Figure 6 depicts the parameter selection for the AAO algorithm for the classification of liver cirrhosis. 

### 3.6. AAO with GMM

The combination of AAO and GMM as a classifier leverages the optimization capabilities of AAO to explore and exploit the search space effectively. GMM, as a classifier, provides a probabilistic framework for modeling and classifying the data based on the extracted features. This hybrid approach aims to improve the performance of classification tasks by incorporating the strengths of both AAO and GMM. The proposed method combines AAO with GMM to obtain the optimum result. It is observed that in Table 2, compared with the harmonic search algorithm, the AAO attains the minimum mean square error (MSE). Hence, the AAO is hybridizing with GMM. 

## 4. Results and Discussion

The classifier’s efficiency is measured using a ten-fold cross-validation procedure, and the measures employed in this work are as follows: if a positive sample is correctly predicted as positive, it is referred to as (TP) true positive; if a negative sample is correctly predicted as negative, it is said to be (TN) true negative. A false positive (FP) occurs when a result is incorrectly assumed to be positive when it is actually negative. A false negative (FN) happens when a result is mistakenly assumed to be negative but is really positive.

The mean square error can be calculated using the formula below:

(22) $$MSE\;=\;\frac{1}{K}\sum_{j=1}^{K}(O_{j}\;-\;T_{i}{)}^{2},$$

where *O_j_* is the computed value at a particular time and *T_i_* is the desired value at system *i*. The training was carried out in such a way that the classifier’s MSE is reduced to a very small value. Table 2 compare the MSE of eight classifiers with three sets of clustering-based features and one entropy-based feature for the classification of normal and cirrhotic liver images. 

From Table 2, it is observed that for PCM feature, AAO, AAO with GMM, and SVM (RBF) classifier provide minimum MSE values of 2.31 × 10^−5^, 2.22 × 10^−5^, and 2.53 × 10^−5^, respectively, at the same time, SVM (RBF) and the AAO classifier lead to type-I (false Positive) error. The classifiers GMM, SDC, HS, SVM (linear), and SVM (polynomial) lead to both type-I and type-II errors (false negative) and obtain maximum MSE values of 1.09 × 10^−4^, 5.19 × 10^−5^, 1.17 × 10^−4^, 3.33 × 10^−4^, and 7.38 × 10^−5^, respectively. Similarly, for the FCM feature, the AAO algorithm provides the least MSE (1.22 × 10^−6^). The classifiers HS and SVM (linear) lead to a type-I error and GMM leads to a type-II error. As a whole, the lower error (1.22 × 10^−5^, 4.57 × 10^−6^, and 1.39 × 10^−6^) was obtained for SVM (polynomial), SVM (RBF), and AAO with GMM classifiers, and the maximum MSE was obtained for GMM, SDC, HS, and SVM (linear); the values are 1.37 × 10^−5^, 5.78 × 10^−5^, 2.40 × 10^−5^, and 3.42 × 10^−4^. For the PFCM feature, again, AAO with GMM hybrid classifier yields minimum MSE of 2.6 × 10^−7^, and HS brings out a type-I error, where SDC leads to a type-II error. So, the classifiers SDC, HS, SVM (linear) result in maximum MSE of 3.701 × 10^−5^, 1.249 × 10^−5^, and 1.306 × 10^−5^; and GMM, SVM (polynomial), SVM (RBF), and AAO bring about lower MSE values of 2.05 × 10^−6^, 8.545 × 10^−6^, 3.145 × 10^−6^, and 5.125 × 10^−6^, respectively. Likely, for the sample entropy feature, the minimum MSE of 8.1 × 10^−7^ was attained for the AAO classifier. The GMM AND SVM (polynomial) classifiers lead to type-I and type-II errors. Lower MSE values of 1.265 × 10^−5^, 1.945 × 10^−6^, and 2.745 × 10^−6^ were attained by SDC, SVM (RBF), and AAO with the GMM classifier. But the classifiers GMM, HS, SVM (linear), and SVM (polynomial) achieved a maximal MSE of 1.331 × 10^−5^, 4.42 × 10^−5^, 5.545 × 10^−5^, 1.702 × 10^−5^, correspondingly.

### 4.1. Selection of Classifier Parameters

The ultrasonic liver dataset consisted of two distinct classes—specifically, cirrhotic and normal—which were used to determine the target values. The target variable was accurately chosen to contain greater values within the range of 0 to 1. The following criterion is applied in order to select the target for the normal case (*T_Norm_*):

(23) $$\frac{1}{K}\;\sum_{i=1}^{K}\mu_{i}\leq \;T_{Norm}.$$

For ‘*K*’ numbers of normal images, the mean for normalized features are represented by $\mu_{i}$, and the value is between 0 and 1. The abnormal case the condition for the target selection is

(24) $$\frac{1}{K}\;\sum_{j=1}^{K}\mu_{j}\;\leq \;T_{Abnnormal}.$$

Here, $\mu_{j}$ represents the mean of normalized features for the abnormal case. For best classification, $\left\|T_{Abnormal}\;-\;T_{Norm}\right\|\;\geq \;0.5$. Considering the above-mentioned statement, the target for this work has been set to 0.1 and 0.85 for normal and abnormal, respectively.

Table 3 shows the trial and error method for selecting the best parameters for the classifier during the training process. For controlling the convergence criteria, the maximum iteration is set to 1000. 

### 4.2. Classifier Performance Analysis

The following parameters are considered to assess the performance of the classifiers. The parameters are accuracy, F1 score, Mathew correlation coefficient (*MCC*), F measure (*FM*), error rate (*ER*), and Jaccard metric (*JM*). Below is a formula that can be used to determine the overall efficiency of the classification method:

(25) $$Accuracy\;(A)\;=\;\frac{TP+TN}{TP+TN+FP+FN}\;\times \;100,$$

(26) $$F1\;Score\;=\;\frac{2TP}{2TP+FP+FN}\;\times \;100,$$

(27) $$\begin{array}{l} \mathrm{Mathew}\;\mathrm{Correlation}\;\mathrm{Coefficient}\;MCC\;=\\ \;\frac{\left(TP\times TN\right)-\;(FP\times FN)}{\sqrt{\left(TP+FP)\;(TP+FN)(TN+FP)(TN+FN\right)}}\;\times 100 \end{array},$$

(28) $$F\;Measure\;=\;\sqrt{\frac{TP}{TP+FP}\;\times \;\frac{TP}{TP+FN}},$$

(29) $$Error\;Rate\;(ER)\;=\;\frac{FN+TP}{TN+TP+FN+F\;P}\;\times \;100,$$

(30) $$Jaccard\;Metric\;(JM)\;=\;\frac{TP}{TP+FP+FN}\;\times 100$$

The performance analysis of the classifiers is exhibited in Table 4 for three cluster-based features and sample entropy feature. As observed in Table 4, the PFCM clustering features with the hybrid classifier AAO–GMM reached a prominent high level in all bench mark parameters, such as an accuracy of 99.03%, an F1 score of 95.24%, an MCC with 0.90, and an FM of 0.95, and achieved a lower error rate of 0.97% with a Jaccard metric of 90.91%. Based on the result, we can now conclude that the hybrid classifier AAO with GMM is the best-performing classifier for the PFCM feature. At the same time, from Table 4, it is observed that the SVM (linear) classifier attained lower-level performance, with an accuracy of 52.38%, an F1 score of 53.49%, a lower MCC value of 0.05, an FM of 0.43, and a higher error rate of 47.62% with JM of 36.51%. Based on the performance, the SVM classifier is the worst-performing classifier in the detection of liver disease detection. By analyzing individual feature analysis, for the PCM feature, the hybrid classifier AAO with GMM performs better than the other classifiers based on an FM of 0.79 and a Jaccard metric of 64.29%. The SVM (linear) is the lower-performing classifier because of its higher error rate of 47.62%, with a reduced MCC of 0.05. Similarly, for the FCM feature, the heuristic classifier AAO attained a best accuracy of 91.67%, with good MCC agreement of 0.83 and a lower error rate of 8.33%. At the same time, for the PFCM feature, the hybrid classifier AAO with GMM attained a higher accuracy of 99.03% because of the lower error rate of 0.97%. In the same way, the AAO classifier for the sample entropy feature achieved a good accuracy of 94.05%, an F1 score of 94.12%, and a good MCC value of 0.88, and this is reduced to lower error rate, which is 5.95%. It is also perceived in Table 4 that when we add AAO with GMM together in cascade form, the performance of the classifier is increased in terms of accuracy, F1 score, MCC, FM, and JM, with a lower error rate. 

**Figure 7:**
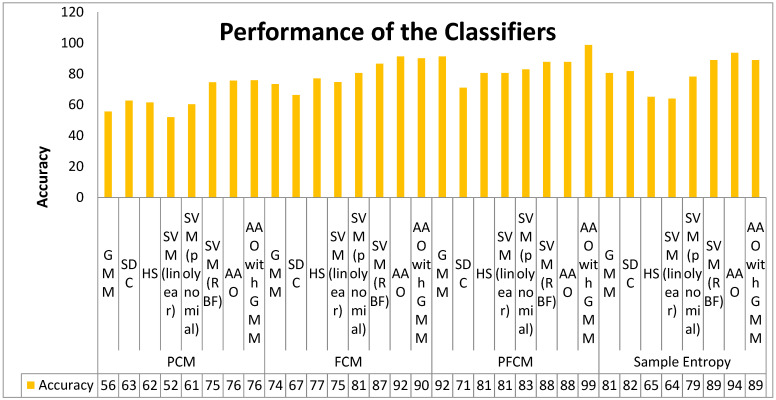
Performance evaluation of different classifiers using cluster and sample entropy features.

Figure 7 depicts the performance indicators, comparing the accuracy of classifiers for PCM, FCM, and PFCM, and sample entropy features for different classifiers.

Table 5 shows the consolidated classifier performance for all the classifiers with four different feature extraction techniques. The hybrid classifier AAO–GMM is the best-performing classifier for the PCM feature, with an accuracy of 76.19%, and the SVM (linear) classifier is the worst-performing classifier for the PCM feature, with an accuracy of 52.38% and a minimum JM of 36.51%. For the PCM feature, the bio-inspired classifier AAO gives good accuracy of 91.67%. with 91.76% F1 score, and lower accuracy obtained by the SDC classifier, with a value of 66.67%. Next, again, the hybrid classifier AAO–GMM attained a very high accuracy of 99.03% for the PFCM feature, with a good F1 score of 95.24%. At the same time, the SDC classifier attained the lowest accuracy (71.43%). For the sample entropy feature, the bio-inspired classifier AAO attained a good accuracy of 94.05%, and lowest (64.29%) was attained by the SVM (linear) classifier. When comparing all the classifiers with four feature extraction techniques, the hybrid classifiers perform better and provide maximum accuracy due to the hybrid nature of GMM and the bio-inspired classifier. Among the eight classifiers, the SVM (linear) and SDC classifiers are the worst-performing classifier due to their non-linear nature and the presence of outliers in the extracted features. 

**Figure 8:**
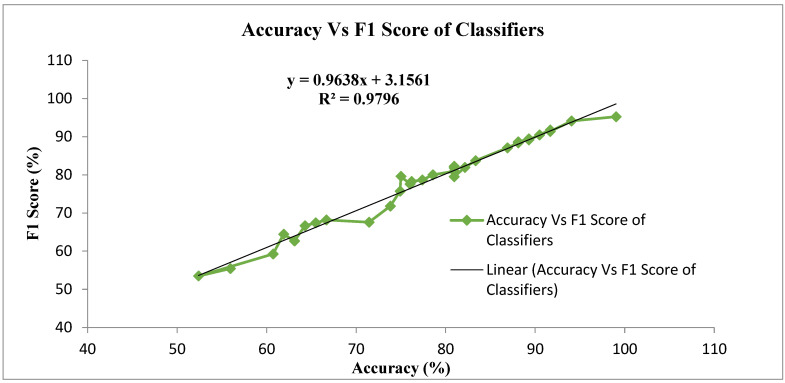
Overall analysis of accuracy vs. F1 score for different classifiers.

Figure 8 shows the overall accuracy and F1 score analysis of the classifiers with the four feature extraction techniques. The linear nature of the classifiers is below 65% and above 85% accuracy in Figure 8. In the range between 65% and 85%, the classifiers exhibit non-linearity.

## 5. Conclusions and Future Scope

The foremost aim of this work is to classify the ultrasonic liver images as normal or cirrhotic in an accurate manner. The features of ultrasonic images are extracted using PCM, FCM, PFCM, and sample entropy methods. There are eight classifiers, namely, GMM, SDC, harmonic search, AAO, SVM (linear), SVM (polynomial), SVM (RBF), AAO, and the hybrid classifier AAO–GMM, employed for classification purposes. The hybrid classifier AAO–GMM for the PFCM feature attained a better accuracy of 99.03%, followed by the sample-entropy-based feature with the AAO classifier, with an accuracy of 94.04%. This proposed method is not applied for patients with cirrhosis who are awaiting liver transplantation. In the future, the proposed methodology will be employed due to improved accuracy in the detection of liver abnormalities using machine learning algorithms. It offers significant potential to improve the prognosis for patients with cirrhosis who are awaiting liver transplantation. These advanced technologies can enhance early therapeutic interventions, optimize surgical planning, and refine transplantation strategies. By enabling earlier and more-precise interventions, optimizing surgical and transplantation decisions, and personalizing treatment plans, these advanced technologies can significantly enhance patient outcomes. Further research will be undertaken in the direction of analyzing deep learning features with CNN, DNN, and LSTM architectures for liver abnormalities with multiple databases.

## Figures and Tables

**Table 1:** Statistical parameters for PCM, FCM, and FPCM and sample entropy features in normal and cirrhotic liver images.

Feature Extraction Method	PCM		FCM		PFCM		Sample Entropy	
Statistical Parameters	Cirrhosis	Normal	Cirrhosis	Normal	Cirrhosis	Normal	Cirrhosis	Normal
Mean	0.5054	0.4968	0.1509	0.1569	0.2828	0.2833	5.339727	5.2504
Variance	0.0468	0.0456	0.0010	0.0014	0.0079	0.0089	0.137262	0.2105
Skewness	−0.2156	−0.1960	−0.6516	−0.764	1.6003	1.7111	−1.75338	−1.6610
Kurtosis	−0.8087	−0.8578	0.7095	0.9353	3.1021	3.1411	10.75434	7.3273
Pearson Correlation coefficient (PCC)	0.0092	−0.0212	0.5839	0.5453	0.3721	0.4374	0.5030	0.4914
Canonical Correlation Analysis (CCA)	0.4190		0.7655		0.7569		0.6046	

**Table 2:** Average MSE and confusion matrix for classifiers based on clustering and sample entropy features.

Feature Extraction	Classifiers	TP	TN	FP	FN	MSE
PCM	GMM	509	531	398	421	1.09 × 10^−4^
	SDC	576	597	332	354	5.19 × 10^−5^
	Harmonic search (HS)	642	509	420	288	1.17 × 10^−4^
	SVM (linear)	509	465	464	421	3.33 × 10^−4^
	SVM (polynomial)	531	597	332	399	7.38 × 10^−5^
	SVM (RBF)	700	640	289	160	2.53 × 10^−5^
	Artificial algae optimization (AAO)	772	641	288	158	2.31 × 10^−5^
	**AAO–GMM**	**797**	**619**	**310**	**133**	**2.22 × 10^−5^**
FCM	GMM	619	752	177	311	3 × 10^−5^
	SDC	664	575	354	266	4.09 × 10^−5^
	Harmonic Search (HS)	774	663	266	156	1.62 × 10^−5^
	SVM (linear)	907	486	443	23	2.01 × 10^−4^
	SVM (polynomial)	752	752	177	178	1.22 × 10^−5^
	SVM (RBF)	818	796	133	112	4.57 × 10^−6^
	**Artificial Algae optimization (AAO)**	**870**	**860**	**69**	**60**	**1.22 × 10^−6^**
	Artificial algae optimization (AAO) with GMM	841	840	89	89	1.39 × 10^−6^
PFCM	GMM	819	885	44	111	2.05 × 10^−6^
	SDC	554	774	155	377	3.701 × 10^−5^
	Harmonic search (HS)	819	686	243	111	1.249 × 10^−5^
	SVM (linear)	797	708	221	133	1.306 × 10^−5^
	SVM (polynomial)	797	752	177	133	8.54 × 10^−6^
	SVM (RBF)	841	796	133	89	3.145 × 10^−6^
	Artificial algae optimization(AAO)	863	774	155	67	5.125 × 10^−6^
	**AAO–GMM**	**886**	**885**	**44**	**44**	**2.6 × 10^−7^**
Sample Entropy	GMM	686	818	111	244	1.331 × 10^−5^
	SDC	753	774	155	177	1.265 × 10^−5^
	Harmonic search (HS)	664	553	376	266	4.42 × 10^−5^
	SVM (linear)	664	531	398	266	5.545 × 10^−5^
	SVM (polynomial)	797	664	265	133	1.702 × 10^−5^
	SVM (RBF)	841	818	111	89	1.945 × 10^−6^
	**Artificial Algae optimization(AAO)**	**886**	**863**	**66**	**44**	**6.5 × 10^−7^**
	AAO–GMM	819	841	88	111	2.745 × 10^−6^

**Table 3:** The selection of optimum parameters for the classifier.

Classifier	Optimal Parameter of the Classifier
Gaussian Mixture Model (GMM)	The mean and covariance of the input samples, as well as the tuning parameter, are estimated using the expectation maximization (EM) algorithm. Criterion: MSE
Softmax discriminant classifier (SDC)	The value of λ is 0.5, and the mean of the target values for each class are 0.1 and 0.85, respectively.
Harmonic search algorithm	Class harmony will always be maintained at the predetermined target values of 0.85 and 0.1. Adjustments are made to the upper and lower bounds using a step size of Δw = 0.005 for each. The final harmony aggregation is achieved when the MSE is less than 10^−5^ or when the maximum iteration count reaches 1000, depending on which comes first. Criterion: MSE
SVM (linear)	C (regularization parameter): 0.85; class weight: 0.4; convergence criterion: MSE
SVM (polynomial)	C = 0.8; kernel function coefficient γ: 10; class weight: 0.5; convergence criterion: MSE
SVM (RBF)	C = 0.8; kernel function coefficient γ: 100; class weight: 0.87; convergence criterion: MSE
Artificial algae optimization (AAO)	Share force: 3; energy loss: 0.4; adaptation: 0.3; convergence criterion: MSE
AAO with GMM	tuning parameter is EM; share force: 3; energy loss: 0.4; adaptation: 0.3; convergence criterion: MSE

**Table 4:** Performance analysis of classifiers for clustering and sample entropy features.

Feature Extraction	Classifiers	Accuracy	F1 Score	MCC	F Measure	ER	JM
PCM	GMM	55.95	55.42	0.12	0.55	44.05	38.33
	SDC	63.10	62.65	0.26	0.63	36.90	45.61
	Harmonic search	61.90	64.44	0.24	0.65	38.10	47.54
	SVM (linear)	52.38	53.49	0.05	0.54	47.62	36.51
	SVM (polynomial)	60.71	59.26	0.21	0.59	39.29	42.11
	SVM (RBF)	74.90	75.72	0.51	0.76	25.10	60.92
	AAO	76.01	77.59	0.53	0.78	23.99	63.38
	**AAO with GMM**	**76.19**	**78.26**	**0.53**	**0.79**	**23.81**	**64.29**
FCM	GMM	73.81	71.79	0.48	0.72	26.19	56.00
	SDC	66.67	68.18	0.33	0.68	33.33	51.72
	Harmonic search	77.38	78.65	0.55	0.79	22.62	64.81
	SVM (linear)	75.00	79.61	0.56	0.81	25.00	66.13
	SVM (polynomial)	80.95	80.95	0.62	0.81	19.05	68.00
	SVM (RBF)	86.90	87.06	0.74	0.87	13.10	77.08
	**AAO**	**91.67**	**91.76**	**0.83**	**0.92**	**8.33**	**84.78**
	AAO with GMM	90.48	90.48	0.81	0.90	9.52	82.61
PFCM	GMM	91.67	91.36	0.84	0.91	8.33	84.09
	SDC	71.43	67.57	0.44	0.68	28.57	51.02
	Harmonic search	80.95	82.22	0.63	0.82	19.05	69.81
	SVM (linear)	80.95	81.82	0.62	0.82	19.05	69.23
	SVM (polynomial)	83.33	83.72	0.67	0.84	16.67	72.00
	SVM (RBF)	88.10	88.37	0.76	0.88	11.90	79.17
	AAO	88.10	88.64	0.77	0.89	11.90	79.59
	**AAO with GMM**	**99.03**	**95.24**	**0.90**	**0.95**	**0.97**	**90.91**
Sample entropy	GMM	80.95	79.49	0.63	0.80	19.05	65.96
	SDC	82.14	81.93	0.64	0.82	17.86	69.39
	Harmonic search	65.48	67.42	0.31	0.68	34.52	50.85
	SVM (linear)	64.29	66.67	0.29	0.67	35.71	50.00
	SVM (polynomial)	78.57	80.00	0.58	0.80	21.43	66.67
	SVM (RBF)	89.29	89.41	0.79	0.89	10.71	80.85
	**AAO**	**94.05**	**94.12**	**0.88**	**0.94**	**5.95**	**88.89**
	AAO with GMM	89.29	89.16	0.79	0.89	10.71	80.43

**Table 5:** Consolidated performance analysis of individual classifiers.

Feature Extraction Technique	Classifiers	Accuracy	F1 Score	JM
PCM	SVM (linear)	52.38	53.49	36.51
	AAO with GMM	76.19	78.26	64.29
FCM	SDC	66.67	68.18	51.72
	AAO	91.67	91.76	84.78
PFCM	SDC	71.43	67.57	51.02
	AAO with GMM	99.03	95.24	90.91
Sample entropy	SVM (linear)	64.29	66.67	50.00
	AAO	94.05	94.12	88.89

## Data Availability

The data that support the findings of this study are available from the corresponding author upon reasonable request.
